# Characterization of the complete chloroplast genome of the medicinal plant *Orixa japonica* (Rutaceae) in Zhejiang Province and its phylogenetic analysis within family Rutaceae

**DOI:** 10.1080/23802359.2021.1931511

**Published:** 2021-05-24

**Authors:** Zhiqi Ying, Meixiu Yan, Manjia Zhou, Xiangyu He, Rubin Cheng

**Affiliations:** College of Pharmaceutical Science, Zhejiang Chinese Medical University, Hangzhou, China

**Keywords:** *Orixa japonica*, Rutaceae, complete chloroplast genome, phylogenetic analysis

## Abstract

*Orixa japonica* Thunb. is an important medicinal plant belonging to the family Rutaceae. In this study, we determined the the complete chloroplast (cp) genome of *O. japonica*, which was 158,525 bp in length containing one large single copy region (85,965 bp), one small single copy region (18,552 bp), and a pair of inverted repeat regions (27,004 bp each). A total of 134 genes were annotated in the cp genome, including 88 protein coding genes, 37 tRNA genes, eight rRNA genes, and one pseudo gene ycf1. According to the phylogenetic analysis, *O. japonica* clustered together with *Casimiroa edulis* with high bootstrap value, indicating a close genetic relationship with subfamily Amyridoideae.

*Orixa japonica* Thunb. (*O. japonica*) is a shrub or tree belonging to the family Rutaceae that has noteworthy medicinal value and is worth doing profound researches. It is widely distributed in southern China (Thunb 1997). The roots and stems of it are used as a kind of herbal medicine in Chinese folk. According to records, it has certain toxicity and can clear heat and dampness, relieve cough, analgesia, and emetic. It is used to treat stomachache, rheumatic arthralgia, malaria, etc. The extracts or constituents of it possess pharmacological and biological activities. Previous chemical studies have isolated and identified several quinoline alkaloids, terpenoids, and coumarins from it and pyrrolidine alkaloid isolated from root bark of the *O. japonica* has potential nematicidal and larvicidal activity that can be developed as natural nematicides and larvicides in the future (Liu et al. 2016). However, the genomic information of *O. japonica* has not been established sufficiently, the complete chloroplast (cp) genome of *O. japonica* has not been sequenced, which limits the in-depth study of it. The complete cp genome contains a lot of molecular information that is a useful tool for phylogenetic analysis and further development on species identification as well as conservation strategies. Therefore, it is worthwhile to sequence the complete cp genome of *O. japonica* and analyze characters of it to pave the way for its further genetics research.

In this study, the complete cp genome of *O. japonica* has been determined and its phylogenetic relationship within family Rutaceae also has been analyzed through the complete cp genome. Total genomic DNA was extracted from fresh leaves of *O. japonica* specimen by modified CTAB method (Doyle and Doyle 1987). The specimen was collected from Fuyang District, Hangzhou City, Zhejiang Province (30°04′59.74″N, 119°53′10.14″E) and deposited in the Medicinal Herbarium Center of Zhejiang Chinese Medical University, Hangzhou, Zhejiang, China (voucher identifying number CCSZK-200816). Illumina Hiseq Platform (Illumina, San Diego, CA) was used to sequence the total genomic DNA and metaSPAdes (Nurk et al. 2017) was used to assemble the complete cp genome of *O. japonica* with the complete cp genome of *Phellodendron amurense* (GenBank accession number: NC_035551) as reference (Dong et al. 2020; Gao et al. 2020). The complete cp genome of *O. japonica* was annotated using Geseq (Tillich et al. 2017) and then was manually confirmed by BLAST. The final annotated cp genome of *O. japonica* was submitted to GenBank with the accession number MW574915.

The complete cp genome of *O. japonica* in total is 158,525 bp in length and consists of a large single copy region (LSC; 85,965 bp), a small single copy region (SSC; 18,552 bp), and a pair of inverted repeat regions (IRs; 27,004 bp each). There are a total of 134 genes annotated in the cp genome, including 88 protein coding genes, 37 tRNA genes, eight rRNA genes, and one pseudo gene *ycf1*. In addition, the overall GC content of the complete cp genome is 38.21%, and the corresponding values for LSC, SSC, and IR regions are 36.49%, 32.68%, and 42.86%, respectively. There are 21 duplicated genes in the IR region.

In order to perform the phylogenetic analysis, a maximum-likelihood (ML) tree was constructed using the complete cp genome sequence of *O. japonica* and 20 other species from family Rutaceae, *Carapa guianensis* was selected as the outgroup. These complete cp genome sequences were downloaded from NCBI and first aligned using MAFFT v7.037b (Katoh and Standley 2013), then the ML tree was constructed by MEGA 7 (Kumar et al. 2016) with 100 bootstrap replications. According to the ML tree (Figure 1), *O. japonica* clustered together with *Casimiroa edulis* with high bootstrap value, indicating that they have close relationship. In *Flora Reipublicae Popularis Sinicae*, genera *Ruta*, *Orixa*, *Melicope*, *Tetradium*, and *Zanthoxylum* belong to the subfamily Rutoideae (Engl 1997a, 1997b), genera *Casimiroa*, *Phellodendron*, and *Toddalia* belong to the subfamily Toddalioideae (Engl 1997a, 1997b). However, in the most recent classification of Rutaceae, Amyridoideae is the most diverse subfamily, genera *Casimiroa*, *Melicope*, *Phellodendron*, *Tetradium*, *Toddalia*, and *Zanthoxylum* belong to the subfamily Amyridoideae (Morton and Telmer 2014; Sun et al. 2021), but which subfamily genus *Orixa* belongs to is still not clear. According to our result, *O. japonica* formed a clade with the plants from subfamily Amyridoideae, suggesting that *O. japonica* might has close genetic relationship with subfamily Amyridoideae. These findings provide fundamental valuable molecular information for *O. japonica* and laid a foundation for its identification, as well as further research on genetics of family Rutaceae in the future.

**Figure 1.**
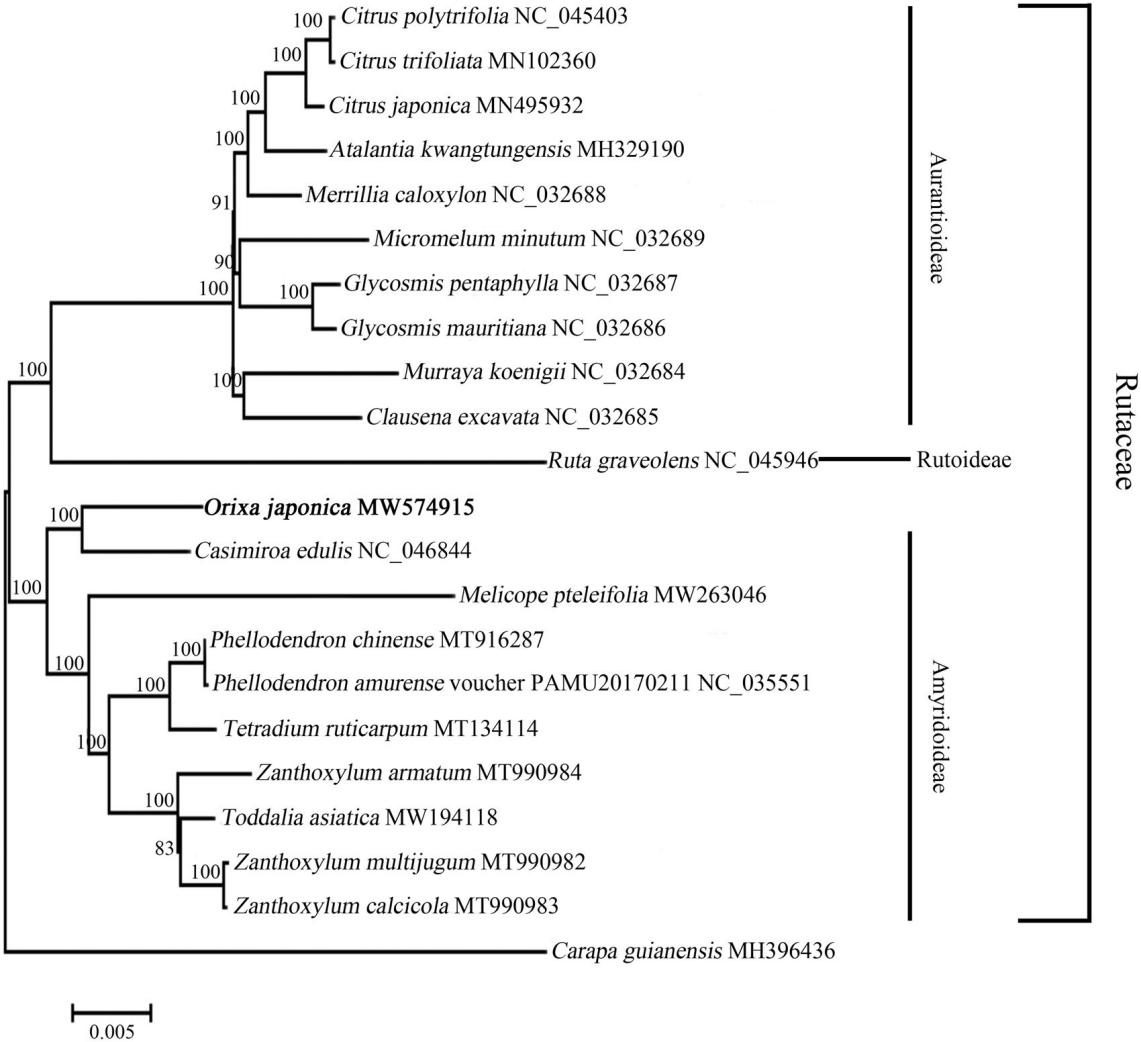
Phylogenetic relationships of *Orixa japonica* and 20 other species from family Rutaceae. The maximum-likelihood (ML) tree was constructed by MEGA 7 based on Kimura 2-parameter model using the complete chloroplast genomes. *Carapa guianensis*, which was from family Meliaceae, was selected as the outgroup. Numbers on the nodes represent bootstrap values from 100 replicates. The GenBank accession numbers were listed following the species name.

## Data Availability

The genome sequence data that support the findings of this study are openly available in GenBank of NCBI at https://www.ncbi.nlm.nih.gov/ under the accession no. MW574915. The associated BioProject, SRA, and BioSample numbers of *Orixa japonica* are PRJNA702044, SRR13717521, and SAMN17922835, respectively.
